# Ischemia-Modified Albumin, a Novel Predictive Marker of In-Hospital Mortality in Acute Aortic Dissection Patients

**DOI:** 10.3389/fphys.2019.01253

**Published:** 2019-09-27

**Authors:** Guifang Yang, Yang Zhou, Huaping He, Xiaogao Pan, Xiangping Chai

**Affiliations:** ^1^Department of Emergency Medicine, The Second Xiangya Hospital, Central South University, Changsha, China; ^2^Emergency Medicine and Difficult Diseases Institute, Central South University, Changsha, China

**Keywords:** acute aortic dissection, ischemia-modified albumin, in-hospital mortality, AAD, IMA

## Abstract

**Background:**

This work explored the prognostic prediction capabilities of ischemia-modified albumin (IMA) in patients suffering from acute aortic dissection (AAD).

**Methods:**

We conducted a retrospective analysis using electronic health records. This study included AAD patients admitted to the Second Xiangya Hospital of Central South University from January 2015 to December 2018 in ≤24 h from the onset of symptoms to hospital admission. The levels of IMA were recorded upon admittance and the final was the all-cause mortality during hospitalization.

**Results:**

This study enrolled 731 AAD patients. Among who, 160 passed away in the course of medication while 571 of them survived. Those who passed away exhibited higher levels of IMA (94.35 ± 26.84 vs. 69.14 ± 14.70, *p* < 0.001) than the survivors. Following the adjustment confounders, the fully adjusted model showed IMA to be an independent forecastor for in-hospital mortality for AAD patients (OR 1.10, 95% CI 1.08–1.13, *p* < 0.001). Analysis based on receiver operating characteristic (ROC) revealed that 79.35 μ/ml was the best threshold of IMA level. The area under the curve (AUC) based on this IMA level was 0.854 (95% CI 0.822–0.898) while the specificity and sensitivity to anticipate in-hospital death were 84.8 and 80.6%, respectively.

**Conclusion:**

Admission IMA was an independent forecastor for in-hospital mortality among people suffering from AAD.

## Introduction

Acute aortic dissection (AAD), a fatal aortic sickness has high death rate and morbidity which demands prompt examination and treatment (Yang et al., 2016; Bossone et al., 2018). From 1 to 2% of patients with AAD die per hour for the first 24–48 h (Milewicz and Ramirez, 2019). Unfortunately, there is still a lack of effective methods to accurately predict the short-term results of these patients. Ischemia-modified albumin (IMA), was recently found out to be a marker for acute myocardial ischemia, plays an important role in the early diagnosis of cardiogenic ischemic diseases (Kontos et al., 2003; Kalay et al., 2007). IMA is a Food and Drug Administration (FDA) approved diagnostic indicator of the early stage of myocardial ischemia among patients having acute coronary syndrome (ACS), which can significantly reduce the missed diagnosis rate in patients with cardiovascular disease (Montagnana et al., 2008). In recent years, many new studies have found that the serum level of IMA can also be significantly increased in non-cardiogenic ischemic diseases (Sbarouni et al., 2006; Bolatkale et al., 2017). Furthermore, IMA has been shown to be an effective marker for the seriousness as well as prognosis of diseases like acute ischemic chest pain patients in emergency department (Consuegra-Sanchez et al., 2008), continuous ambulatory peritoneal dialysis patients (Su et al., 2013), and severe sepsis patients (Yin et al., 2017). However, there is no predictive value for of IMA in the prognosis of AAD patients has not yet been reported. Thus, this work explored the association of IMA and in-hospital mortality of AAD patients.

## Materials and Methods

### Research Design and Settings

This is a retrospective study design. Medical records of AAD patients in the Second Xiangya Hospital of Central South University from January 2015 to December 2018 were investigated. This research was examined and authorized by the hospital ethics committee, being a retrospective study, informed consent was waived.

The classification of AAD was done based on Stanford criteria while magnetic resonance angiography (MRA) or computed tomography angiography (CTA) (Erbel et al., 2015) were used to confirm the diagnosis of AAD. This study included AAD patients who were admitted in ≤24 h from the time of showing symptoms. Exclusion criteria included: (1) uncompleted IMA tests; (2) prior history of malignant tumor or liver cirrhosis; (3) diagnosis with pregnancy; (4) the time of admission was ≥24 h from the onset of symptoms.

### Measurement of Serum IMA Levels

Samples of patients’ blood were collected from the brachial veins and put into plastic Vacutainer tubes covered with gel for measurement of IMA levels. Centrifugation was carried out for 15 min at 1620 *g* to separate the serum, decantation was then carried out and IMA measurements were immediately as per the manufacturer’s instructions via albumin cobalt test kit (Yikang Science Technique Development Co., Changsha, China).

### AAD Treatment

Urapidil, nitroglycerin or sodium nitroprusside was injected into AAD patients combined who had high blood pressure to maintain the systolic blood pressure (SBP) at 100–120 mm Hg. All patients were given beta blockers except for contraindications. Patients with acute type A aortic dissection and a minimal number of patients with acute type B aortic dissection were surgically repaired under cardiopulmonary bypass. Under general anesthesia, endovascular repair was performed on acute type B aortic dissection patients using available grafts. AAD Patients who did not undergo surgery were given conservative medical treatment (Nienaber and Clough, 2015).

### Variables Included for Analysis

During admission: the age, body mass index (BMI), SBP, diastolic blood pressure (DBP), gender, the presence of hypertension, diabetes, stroke, atherosclerosis, marfan syndrome, chronic renal insufficiency (CRI), and smoking, symptom, myocardial ischemia data were recorded. Type of AAD (Stanford), laboratory data including IMA; hemoglobin (Hb); alanine transaminase (ALT); aspartate aminotransferase (AST); albumin (ALB); total bilirubin (TB); direct bilirubin (DB); creatinine (Cr), Troponin T (cTnT), creatine kinase Mb (CK-Mb) and management were collected subsequently.

### Clinical Endpoint

The clinical study was terminated upon death during hospitalization.

### Statistical Analysis

Statistical data was presented in mean ± standard deviation for normal data while non-normal data, interquartile range (IQR) and median were used. The categorical variables were presented as percentage and number. Wilcoxon Mann–Whitney tests for non-normally distributed continuous variables and unpaired Student’s *t*-tests for normally distributed continuous variables were used to establish the correlations among the survivor and the non-survivor groups. Categorical variables were analyzed using Fisher’s exact test or chi-squared test. To identify the risk factors for in-hospital mortality among AAD patients, analysis was carried out using multivariate regression. We constructed three models, which namely: crude model, with no adjustment of covariates; model I, adjusted for socio-demographic data; model II, model I including other covariates presented in Table 1 were constructed. Sensitivity analysis was carried out for robustness during data analysis. IMA was converted to an absolute variable and *P*-value for tendency was determined. The optimal threshold with high specificity and sensitivity for IMA to forecast in-hospital mortality was detected using receiver operating characteristic (ROC) analysis. It was found out that all the values of *P* were two-sided, and a *P* < 0.05 was considered to be statistically significant. R^1^ and Empower states^2^, X&Y Solution, Inc., Boston, MA) software were used to carry out all the statistical analyses.

**TABLE 1:** Basline characteristics of the patients.

**Variable**	**All patients (*n* = 731)**	**Survivor (*n* = 571)**	**Non-survivor (*n* = 160)**	***P***
Age(years)	52.99 ± 12.17	52.87 ± 11.91	53.41 ± 13.12	0.619
BMI(kg/m^2^)	25.13 ± 4.63	25.21 ± 4.47	24.86 ± 5.16	0.393
SBP(mmHg)	145.61 ± 30.30	148.18 ± 28.66	136.44 ± 34.11	<0.001
DBP(mmHg)	81.22 ± 18.52	83.14 ± 17.88	74.38 ± 19.17	<0.001
IMA(μ/ml)	74.66 ± 20.84	69.14 ± 14.70	94.35 ± 26.84	<0.001
Hb(g/L)	125.05 ± 20.98	126.04 ± 19.84	121.55 ± 24.37	0.017
ALT(μ/L)	20.50 (14.00–36.70)	19.80 (13.30–34.80)	23.20 (15.43–56.50)	<0.001
AST(μ/L)	21.10 (16.00–34.50)	20.30 (15.60–30.40)	24.80 (17.78–75.65)	<0.001
ALB(g/L)	35.62 ± 4.61	35.84 ± 4.55	34.84 ± 4.76	0.016
TB(μmol/L)	14.60 (9.80–20.40)	14.50 (9.85–20.20)	14.70 (9.67–22.05)	0.982
DB(μmol/L)	5.30 (3.70–7.80)	5.30 (3.70–7.55)	5.40 (3.68–8.30)	0.313
Cr(μmol/L)	81.50 (65.50–116.60)	78.20 (63.90–101.35)	104.80 (71.15–170.25)	<0.001
cTnT(pg/ml)	8.94 (4.21–21.07)	8.11 (4.14–16.41)	17.04 (4.86–65.45)	<0.001
CK-Mb(μ/L)	12.90 (8.85–18.15)	12.70 (9.00–17.30)	14.70 (8.28–23.83)	<0.001
Gender				0.259
Male	585 (80.03%)	462 (80.91%)	123 (76.88%)	
Female	146 (19.97%)	109 (19.09%)	37 (23.12%)	
Hypertension				0.609
No	221 (30.23%)	170 (29.77%)	51 (31.87%)	
Yes	510 (69.77%)	401 (70.23%)	109 (68.13%)	
Diabetes				0.449
No	702 (96.03%)	550 (96.32%)	152 (95.00%)	
Yes	29 (3.97%)	21 (3.68%)	8 (5.00%)	
Stroke				0.952
No	703 (96.17%)	549 (96.15%)	154 (96.25%)	
Yes	28 (3.83%)	22 (3.85%)	6 (3.75%)	
Atherosclerosis				0.469
No	664 (90.83%)	521 (91.24%)	143 (89.38%)	
Yes	67 (9.17%)	50 (8.76%)	17 (10.62%)	
Marfan syndrome				0.234
No	713 (97.54%)	559 (97.90%)	154 (96.25%)	
Yes	18 (2.46%)	12 (2.10%)	6 (3.75%)	
CRI				<0.001
No	704 (96.31%)	558 (97.72%)	146 (91.25%)	
Yes	27 (3.69%)	13 (2.28%)	14 (8.75%)	
Smoking				0.027
No	510 (69.77%)	387 (67.78%)	123 (76.88%)	
Yes	221 (30.23%)	184 (32.22%)	37 (23.12%)	
Symptom				<0.001
Chest pain	573 (78.39%)	446 (78.11%)	127 (79.38%)	
Back pain	30 (4.10%)	23 (4.03%)	7 (4.38%)	
Abdominal pain	49 (6.70%)	42 (7.36%)	7 (4.38%)	
Syncope	7 (0.96%)	1 (0.18%)	6 (3.75%)	
Other	72 (9.85%)	59 (10.33%)	13 (8.12%)	
Myocardial ischemia				<0.001
No	666 (91.11%)	538 (94.22%)	128 (80.00%)	
Yes	65 (8.89%)	33 (5.78%)	32 (20.00%)	
Type of AAD (Stanford)				<0.001
A	337 (46.10%)	204 (35.73%)	133 (83.12%)	
B	394 (53.90%)	367 (64.27%)	27 (16.88%)	
Management				<0.001
Medical	215 (29.41%)	90 (15.76%)	125 (78.12%)	
Endovascular	310 (42.41%)	303 (53.06%)	7 (4.38%)	
Surgical	206 (28.18%)	178 (31.17%)	28 (17.50%)	

*ALB, albumin; ALT, alanine transaminase; AST, aspartate aminotransferase; BMI, body mass index; CK-Mb, creatine kinase Mb; Cr, creatinine; CRI, chronic renal insufficiency; cTnT, Troponin T; DB, direct bilirubin; DBP, diastole blood pressure; Hb, hemoglobin; IMA, ischemia-modified albumin; SBP, systolic blood pressure; TB, total bilirubin.*

## Results

### Study Population

**FIGURE 1:**
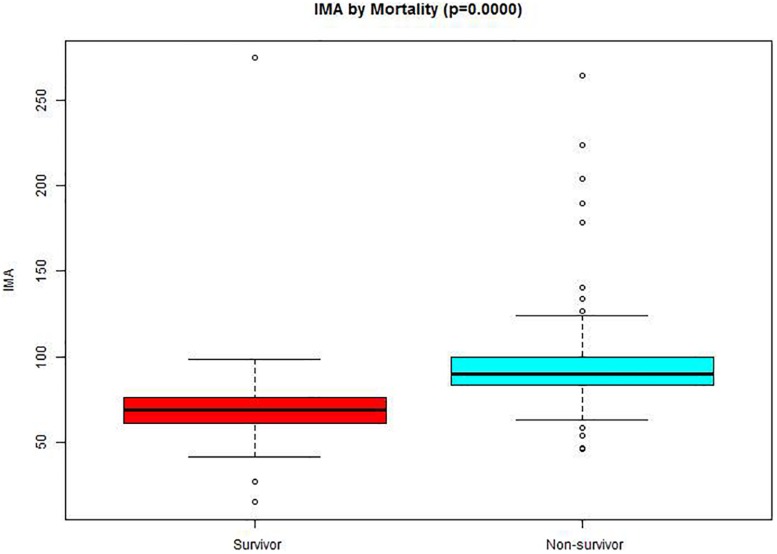
Comparison of IMA values between survival and non-survivor groups. The bottom and top edges of each box represent the first and third quartiles, respectively, the band within the box represents the median value.

A total of 1526 patients were identified with a diagnosis of AAD. Among them, 638 patients uncompleted IMA tests. Nearly all clinical factors were similar in patients with available data on IMA and the 638 patients with missing data on IMA (Supplementary Table S1). 5 patients who had an history of a malignant tumor, 13 patients had a history of liver cirrhosis while 11 patients were expectant. 128 patients got admitted after 24 h of symptoms origin of were excluded from the analysis. 731 AAD patients were included in this study, of which 160 patients passed away during hospitalization while 571 of them survived. Table 1 shows the details of the patients. The differences in age, BMI, TB, DB, gender, presence of hypertension, diabetes, stroke, atherosclerosis, and marfan syndrome were not significant among the two groups. The non-survivors group had significantly lower SBP (136.44 ± 34.11 vs. 148.18 ± 28.66, *p* < 0.001), DBP (74.38 ± 19.17 vs. 83.14 ± 17.88, *p* < 0.001), Hb (121.55 ± 24.37 vs. 126.04 ± 19.84), ALB (34.84 ± 4.76 vs. 35.84 ± 4.55) cTnT [17.04 (4.86–65.45) vs. 8.11 (4.14–16.41), *p* < 0.001] and CK-Mb [14.70 (8.28–23.83) vs. 12.70 (9.00–17.30), *p* < 0.001] in comparison with survivors. The non-survivors exhibited less smoking (76.88 against 67.78%, *p* = 0.027), abdominal pain (4.38% against 7.36%, *p* < 0.001) and other symptom (8.12% against 10.33%, *p* < 0.001) with an increased percentage of symptoms of chest pain (79.38% against 78.11%, *p* < 0.001), back pain (4.38% against 4.03%, *p* < 0.001), syncope (3.75% against 0.18%, *p* < 0.001), myocardial ischemia (20.00% vs. 5.78%, *p* < 0.001), type A AAD (83.12% vs. 35.73%, *p* < 0.001) and medical management (78.12% vs. 15.76%, *p* < 0.001). In addition, the levels IMA (94.35 ± 26.84 vs. 69.14 ± 14.70, *p* < 0.001) were increased in non-survivor group than in the survivor group (Figure 1), as well as ALT [23.20 (15.43–56.50) vs. 19.80 (13.30–34.80), *p* < 0.001], AST [24.80 (17.78–75.65) vs. 20.30 (15.60–30.40), *p* < 0.001], Cr [104.80 (71.15–170.25) vs. 78.20 (63.90–101.35), *p* < 0.001]. Meanwhile, the percentage of Endovascular (4.38% vs. 53.06%, *p* < 0.001) and Surgical (17.50% vs. 31.17%, *p* < 0.001) was lower in non-survivor group than in survivor group.

### Results of Unadjusted and Adjusted

In this study, we constructed three models to analyze the independent effects of IMA on in-hospital mortality (univariate and multivariate). The effect sizes (OR) and 95% confidence intervals were listed in Table 2. In the unadjusted model (crude model), the model-based effect size can be explained as the difference in 1 μ/ml of IMA associated with in-hospital mortality (1.12, 95% CI 1.09–1.14). In the minimum-adjusted model (model I), the IMA was increased by 1 μ/ml, in-hospital mortality increased by 12% (1.12, 95% CI 1.10–1.14). In the completely-adjusted model (model II) (harmonized the covariates in Table 1) for each additional 1 μ/ml of IMA, in-hospital mortality increased by 10% (1.10, 95% CI 1.08–1.13). For sensitivity analysis, IMA was converted from continuous to categorical variable (Tertile of IMA), the *P* for trend of IMA with categorical variables in the fully adjusted model was consistent with the result when IMA is a continuous variable.

**TABLE 2:** Relationship between IMA and in-hospital mortality in different models.

**Exposure**	**Crude Model (OR,95CI,*P*)**	**Model I (OR,95CI,*P*)**	**Model II (OR,95CI,*P*)**
IMA(μ/ml)	1.12 (1.09, 1.14) < 0.001	1.12 (1.10, 1.14) < 0.001	1.10 (1.08, 1.13) < 0.001
**IMA (tertile)**			
T1(15.2–66.2)	Ref	Ref	Ref
T2(66.3–78.2)	1.73 (0.78, 3.86) = 0.181	1.72 (0.77, 3.84) = 0.187	2.17 (0.75, 6.32) = 0.153
T3(78.3–274.5)	27.80 (14.07, 54.94) < 0.001	28.35 (14.31, 56.16) < 0.001	44.93 (16.53, 122.17) < 0.001
*P* for trend	<0.001	<0.001	<0.001

*Model I adjusted for age, gender and BMI. Model II adjusted for age, gender, BMI, hypertension, diabetes, stroke, atherosclerosis, Marfan syndrome, CRI, smoking, symptom, Myocardial ischemia, Hb, ALT, AST, ALB, TB, DB, Cr, cTnT, CK-Mb, type of AAD (Stanford) and management. ALB, albumin; ALT, alanine transaminase; AST, aspartate aminotransferase; BMI, body mass index; CK-Mb, creatine kinase Mb; Cr, creatinine; CRI, chronic renal insufficiency; cTnT, Troponin T; DB, direct bilirubin; DBP, diastole blood pressure; Hb, hemoglobin; IMA, ischemia-modified albumin; SBP, systolic blood pressure; TB, total bilirubin.*

### ROC Analysis

**FIGURE 2:**
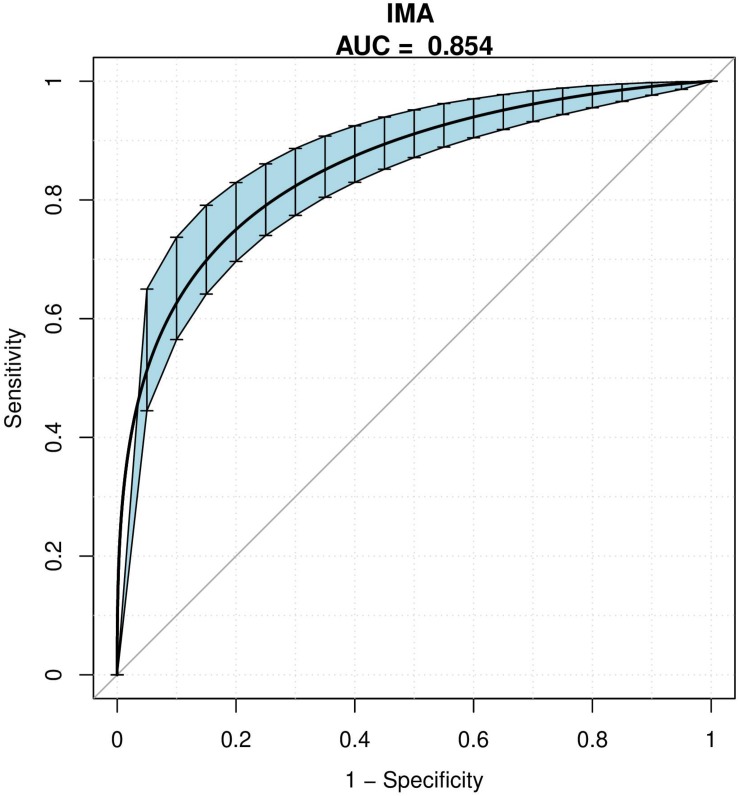
Receiver operating characteristic curves. The AUC value of IMA for predicting in-hospital mortality. Blue shading shows the bootstrap estimated 95% CI with AUC.

The AUC of ROC analysis was 0.854 (95% CI 0.822–0.898). The optimal threshold for IMA to anticipate in-hospital mortality in AAD patients was 79.35 (sensitivity 80.6%, specificity 84.8%) (Table 3 and Figure 2).

**TABLE 3:** Diagnostic value of IMA for in-hospital mortality.

	**ROC area(AUC)**	**95%CI low**	**95%CI up**	**Best threshold**	**Specificity**	**Sensitivity**
IMA(μ/ml)	0.854	0.822	0.898	79.35	0.848	0.806

*CI, confidence interval; IMA, ischemia-modified albumin; OR, odds ratio.*

## Discussion

In this work, IMA levels were shown for the first time to be undoubtedly associated with in-hospital mortality among patients with AAD. Regression analysis identified increased IMA levels were an independent prognosticator for in-hospital mortality. When IMA levels were ≥79.35 μ/L, the specificity, as well as sensitivity for in-hospital mortality, were respectively, 80.6 and 84.8%.

Ischemia-reperfusion, as well as oxidative stress, can be predicted by IMA in various clinical conditions (Borderie et al., 2004). The N-terminus of albumin is damaged under ischemia, and this causes IMA. This has been reported under endothelial damage resulting from extracellular hypoxia, free radical damage, acidosis, and Na-K pump malfunction. Under acute ischemia, the copper, nickel, and cobalt transition metals metal-binding capacity of albumin lower, causing a metabolic variation of the protein, and this is called IMA (Uygun et al., 2011). The existence of ischemia before the development of necrosis can be determined using the IMA test. IMA cannot bind metals at the N-terminus. During album cobalt binding (ACB) test, a predetermined quantity of cobalt is supplemented to the serum sample. The unbound cobalt is determined spectrophotometrically and is proportional to the amount of IMA in the sample (Luo et al., 2014; Eom et al., 2014). Some studies have shown that in the setting of ACS, IMA rises within 30 min and continues to increase for the next 6–12 h, after which its level returns to normal within 24 h (Su et al., 2013).

Currently, there are few studies on the prognostic value of IMA. Worster et al. (2005) revealed that the concentrations of IMA in the first hours after the origin of symptoms have the ability to forecast short as well as long-term results in ACS patients. Further, IMA tested in 6 h after the last bout of chest pain was not a good anticipator for detrimental results. A recent report documented outcomes in a group of 117 patients having critical sepsis. This study showed that the IMA level was lower among survivors than among the non-survivors. It was a powerful forecastor for death after 28-days. An IMA level of greater than 110 μ/ml, could be a good prognosticator of death for patients having severe sepsis (Yin et al., 2017). IMA level in Serum determines the seriousness of systemic deficiency among patients admitted for surgery (Satoh et al., 2014) and could foretell results with end-stage renal disease (Sharma et al., 2006). There is a need for more studies on the prognostic relevance in other acute diseases, mostly those associated with high death rates in their initial phase.

Eroglu et al. (2014) reported that the level of IMA among patients having aortic dissection was more than in healthy individuals. Several mechanisms can be employed to support this concept. During aortic dissection, the arteries from the aorta could be partially or fully obstructed. Cerebrovascular manifestations, limb ischemia, recurrent abdominal pain or renal failure are common as a result of the involvement of a side-branch orifice into the dissection. Moreover, patients with AAD may be hemodynamically unstable with generalized tissue hypoxia, which could also contribute to elevated IMA levels (Sbarouni et al., 2011). However, it was found by Sbarouni et al. (2010) that the values of IMA was 93 ± 19 μ/ml in AAD patients and it is not increased at admission nor increases following surgical repair which is different from our research. This could have been due to the admission of patients at 23 ± 17 h from the beginning of symptom and thus missing the opportune window for detection of IMA. In addition, the number of patients and the research population is different. In our study, 731 AAD patients completed the IMA test, and they had only 46 patients with AAD. In addition, IMA distribution levels in different races have been investigated (blacks and Caucasians), but whether there are differences between other races have not been reported in the literature. Furthermore, the determinants of the performance of the albumin cobalt binding assay are not fully studied and different detection methods may have different results.

Research is dedicated to developing techniques with high sensitivity and specificity for on time prediction of short-term results in AAD patients. However, there is no report on the association of IMA and clinical results in AAD patients. It was revealed for the first time in this study that high IMA levels are contributing to high in-hospital mortality of AAD patients. IMA could be a favorable indicator of risk variation in patients with AAD. The level of IMA does not contribute directly to bad results, it is an indicator of other “upstream” pathologic mechanisms. Resolving the initial disturbance, like safeguarding tissue perfusion and repairing tissue ischemia, might be a good way to decrease IMA levels.

### Limitations of the Study

There were some limitations in this study. For instance, the period of collecting the initial samples of blood was not standardized for each patient and the number of included patients was limited. This was a retrospective study and would be a handicap when finding causal clarifications. The results were based on Chinese subjects and it is unclear whether studies focusing on other nationalities would give related findings.

## Conclusion

Overall, this work established that high levels of IMA were an independent forecastor of in-hospital mortality of AAD patients. IMA can be used as a simple indicator for identifying high-risk AAD patients.

## Data Availability Statement

Datasets used and/or analyzed in the present study were availed by the corresponding author on reasonable request.

## Ethics Statement

The study was approved by the Ethics Committee of the Second Xiangya Hospital of Central South University (Changsha, China) and informed consent was waived due to its retrospective nature.

## Author Contributions

GY wrote the manuscript and recorded the patient’s information. YZ, HH, and XP helped in data collection. XC analyzed and interpreted the patients’ general indices. All authors read and approved the final manuscript.

## Conflict of Interest

The authors declare that the research was conducted in the absence of any commercial or financial relationships that could be construed as a potential conflict of interest.

## Footnotes

1 http://www.R-project.org

2 http://www.empowerstates.com
