# Supplementary material for: Ischemia-Modified Albumin, a Novel Predictive Marker of In-Hospital Mortality in Acute Aortic Dissection Patients
Source: Front Physiol. 2019 Sep 27;10:1253. doi: 10.3389/fphys.2019.01253 (PMC6798049; doi:10.3389/fphys.2019.01253)
Supplement: Supplementary file 1 [file Table_1.DOCX]

**Supplementary table 1. Sensitivity comparative analysis between patients with vs. without Known IMA Data.**

| Variable | Patients with missing IMA data N= 638 | Patients with known IMA N= 731 | P-value |
| --- | --- | --- | --- |
| Age(years) | 52.24 ± 11.70 | 52.99 ± 12.17 | 0.248 |
| BMI(Kg/m^2^) | 24.95 ± 3.98 | 25.13 ± 4.63 | 0.420 |
| SBP(mmHg) | 146.17 ± 27.52 | 145.61 ± 30.30 | 0.722 |
| DBP(mmHg) | 82.05 ± 17.67 | 81.22 ± 18.52 | 0.401 |
| Hb(g/L) | 124.42 ± 21.05 | 125.05 ± 20.98 | 0.575 |
| ALT(u/L) | 21.05 (13.80-39.30) | 20.50 ( 14.00- 36.70) | 0.922 |
| AST(u/L) | 21.00 (15.90-33.08) | 21.10 ( 16.00- 34.50) | 0.561 |
| ALB(g/L) | 35.43 ± 4.61 | 35.62 ± 4.61 | 0.446 |
| TB(umol/L) | 15.10 (9.62-21.40) | 14.60 ( 9.80- 20.40) | 0.923 |
| DB(umol/L) | 5.60 (3.60-8.38) | 5.30 ( 3.70- 7.80) | 0.283 |
| Cr(umol/L) | 82.50 (66.12-109.90) | 81.50 ( 65.50-116.60) | 0.440 |
| cTnT(pg/ml) | 7.98 (3.06-17.11) | 8.94 ( 4.21- 21.07) | 0.569 |
| CK-Mb(u/L) | 11.30 (6.82-16.80) | 12.90 ( 8.85- 18.15) | 0.061 |
| Gender |  |  | 0.967 |
| Male | 510 (79.94%) | 585 (80.03%) |  |
| Female | 128 (20.06%) | 146 (19.97%) |  |
| Hypertension |  |  | 0.490 |
| no | 182 (28.53%) | 221 (30.23%) |  |
| yes | 456 (71.47%) | 510 (69.77%) |  |
| Diabetes |  |  | 0.408 |
| no | 618 (96.87%) | 702 (96.03%) |  |
| yes | 20 ( 3.13%) | 29 ( 3.97%) |  |
| Stroke |  |  | 0.706 |
| no | 611 (95.77%) | 703 (96.17%) |  |
| yes | 27 ( 4.23%) | 28 ( 3.83%) |  |
| Atherosclerosis |  |  | 0.575 |
| no | 585 (91.69%) | 664 (90.83%) |  |
| yes | 53 ( 8.31%) | 67 ( 9.17%) |  |
| Marfan syndrome |  |  | 0.463 |
| no | 626 (98.12%) | 713 (97.54%) |  |
| yes | 12 ( 1.88%) | 18 ( 2.46%) |  |
| CRI |  |  | 0.282 |
| no | 621 (97.34%) | 704 (96.31%) |  |
| yes | 17 ( 2.66%) | 27 ( 3.69%) |  |
| Smoking |  |  | 0.259 |
| no | 427 (66.93%) | 510 (69.77%) |  |
| yes | 211 (33.07%) | 221 (30.23%) |  |
| Symptom |  |  | 0.532 |
| Chest pain | 483 (75.71%) | 573 (78.39%) |  |
| Back pain | 26 ( 4.08%) | 30 ( 4.10%) |  |
| Abdominal pain | 50 ( 7.84%) | 49 ( 6.70%) |  |
| Syncope | 12 ( 1.88%) | 7 ( 0.96%) |  |
| Other | 67 (10.50%) | 72 ( 9.85%) |  |
| Myocardial ischemia |  |  |  |
| no | 598 (93.73%) | 666 (91.11%) | 0.069 |
| yes | 40 ( 6.27%) | 65 ( 8.89%) |  |
|  |  |  |  |
|  |  |  |  |
|  |  |  |  |
| Type of AAD (Stanford) |  |  | 0.607 |
| A | 303 (47.49%) | 337 (46.10%) |  |
| B | 335 (52.51%) | 394 (53.90%) |  |
| Management |  |  | 0.128 |
| Medical | 164 (25.71%) | 215 (29.41%) |  |
| Endovascular | 265 (41.54%) | 310 (42.41%) |  |
| Surgical | 209 (32.76%) | 206 (28.18%) |  |
| Mortality |  |  | 0.297 |
| Survivor | 513 (80.41%) | 571 (78.11%) |  |
| Non-survivor | 125 (19.59%) | 160 (21.89%) |  |

Abbreviations: BMI, body mass index; SBP, systolic blood pressure; DBP, diastole blood pressure; IMA, ischemia-modified albumin; Hb, hemoglobin; ALT, alanine transaminase; AST, aspartate aminotransferase; ALB, albumin; TB, total bilirubin; DB, direct bilirubin; Cr, creatinine; cTnT, Troponin T; CK-Mb, creatine kinase Mb; CRI, chronic renal insufficiency.
